# A systematic review of risk factors for vascular dementia using Mendelian randomization: a call for methodologically robust studies to advance knowledge

**DOI:** 10.1186/s12883-026-04907-4

**Published:** 2026-04-21

**Authors:** Melody Zuo, Sarah A. Gagliano Taliun

**Affiliations:** 1 Faculty of Medicine, Université de Montréal, Montréal, Québec H3C 3J7 Canada; 2 Research Centre, Montréal Heart Institute, 5000 rue Bélanger, Montréal, Québec H1T 1C8 Canada; 3 Department of Medicine, Faculty of Medicine, Université de Montréal, Montréal, Québec H3C 3J7 Canada; 4 Department of Neurosciences, Faculty of Medicine, Université de Montréal, Montréal, Québec H3C 3J7 Canada

**Keywords:** Vascular cognitive impairment, Vascular dementia, Mendelian randomization, Systematic review, Risk factors

## Abstract

**Background:**

Vascular cognitive impairment, of which vascular dementia is the most extreme end of the spectrum, is a result of many risk factors (exposures). Mendelian randomization is a genetic epidemiological approach to assess the causal relationship between a genetically predicted exposure with an outcome of interest that has gained traction in recent years in part due to the emergence of biobank-scale datasets.

**Methods:**

Here, we performed a systematic review of published Mendelian randomization studies to understand the current knowledge of potentially causal genetically predicted exposures on vascular cognitive impairment or vascular dementia risk.

**Results:**

No results were obtained for vascular cognitive impairment. 26 of the 53 of the articles (49%) identified in our review for vascular dementia found evidence using Mendelian randomization for a causal relationship between a genetically predicted exposure and vascular dementia. Among the studies that investigated similar exposures, some consistently yielded positive causal results between certain types of genetically predicted exposures (e.g. predicted markers of inflammation or atherosclerotic vascular disease) and vascular dementia. In contrast, the presence of a causal effect between certain exposures (e.g. cholesterol levels) and vascular dementia was inconsistent depending on the study. Only 10 of the 26 articles (38%) suggesting a causal link included a completed STROBE-MR checklist, warranting caution in interpretation of results.

**Conclusions:**

From our systematic review, we identified evidence of potential causal effects between genetically predicted exposures, such as atrial fibrillation, major depressive disorder and gut microbiota, on vascular dementia risk, some of which are supported by multiple studies whereas others require replication. We strongly encourage future Mendelian randomization studies to follow and report the appropriate checklist (STROBE-MR) to employ triangulation and to include individuals of diverse genetic ancestries to allow for more robust exposures on vascular dementia risk for follow-up.

**Supplementary Information:**

The online version contains supplementary material available at 10.1186/s12883-026-04907-4.

## Introduction

The manifestation of vascular cognitive impairment ranges from mild impairment to dementia associated with cerebrovascular disease. Vascular dementia is the second most common form of dementia after Alzheimer’s disease, resulting in important health implications. 60–70% of dementia cases are attributed to Alzheimer’s whereas vascular dementia accounts for 15–20%, and the prevalence of both increase with age [1]. Non-modifiable risk factors (e.g. sex, age and genetics) and modifiable risk factors (e.g. education, stroke, sleep disorders, depression and lifestyle factors such as diet, physical education and smoking and alcohol use) have been identified as contributors to vascular dementia [2]. Identifying additional risk factors for vascular dementia will be critical to reducing this burden by providing opportunities to delay or prevent disease onset or progression.

Mendelian randomization is an epidemiological approach, which, given the validity of certain assumptions, tests for a causal relationship between a genetically predicted exposure (e.g. a modifiable risk factor) and an outcome (e.g. a disease) using genetic variant-exposure and genetic-variant-outcome statistical associations [3]. It can be a valuable approach for identifying risk factors with causal effects for multifactorial conditions.

The principal objective of this study was to carry out a systematic review of published Mendelian randomization studies that test for a causal link between at least one genetically predicted exposure and vascular dementia to report on the current knowledge of risk factors for this health outcome that may warrant further investigation. Our secondary aim was to provide recommendations for further research to foster discoveries in the field in light of findings from the review.

## Methods

We followed PRISMA reporting guidelines (Additional File 5- Checklist).

A PubMed search was carried out on 7 April 2025 applying the following search term: ((“vascular cognitive impairment“[Title/Abstract] OR “vascular dementia“[Title/Abstract]) AND (“genetic instrumental“[Title/Abstract] OR “genetic instrument“[Title/Abstract] OR “Mendelian randomization“[Title/Abstract] OR “Mendelian randomisation“[Title/Abstract] OR “instrumental variable“[Title/Abstract])) We then applied filters for: Article Language = English and Species = Humans.

We subsequently ensured that none of the articles were labelled as Article Type = “Preprint” or “Retracted Publication” or “Retraction of Publication”. Preprints were excluded as they have not yet undergone peer-review.

As shown above, we searched for either vascular cognitive impairment or vascular dementia. However, as we obtained no results for vascular cognitive impairment we subsequently only refer to vascular dementia as the condition of interest for precision.

The search was carried out independently by two authors. Figures were created using either Microsoft Excel, version 16.98 or Microsoft PowerPoint, version 16.99.1.

## Results

**Fig. 1:**
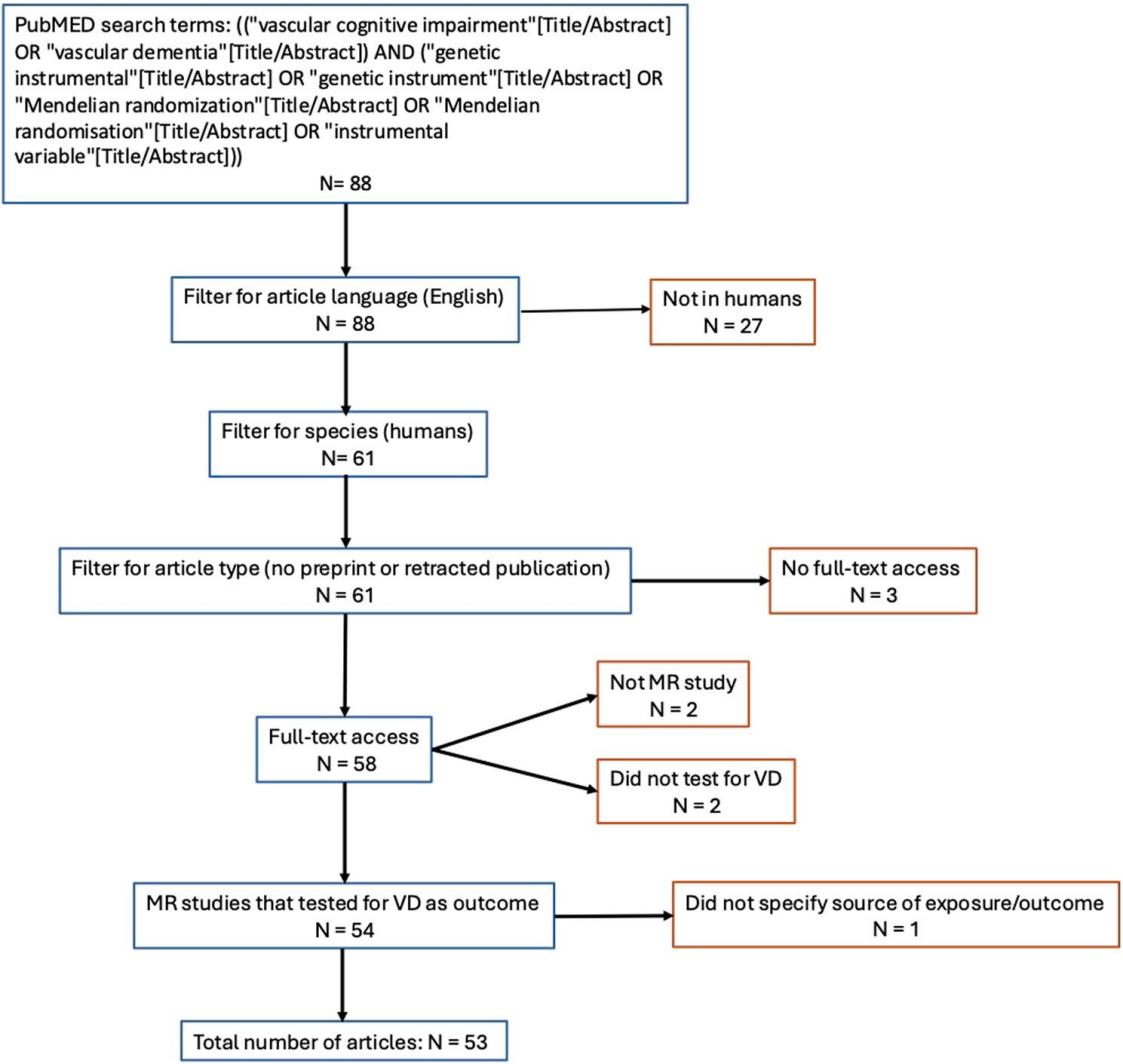
Flowchart of search term and subsequent filtering of articles for the systematic review. VD = vascular dementia, MR = Mendelian Randomization

**Fig. 2:**
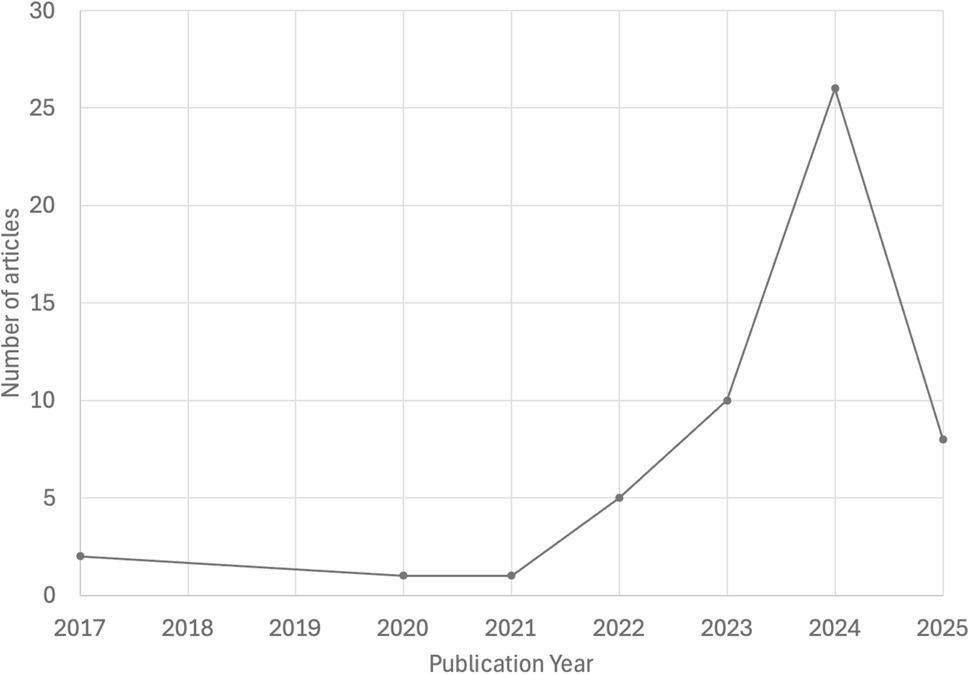
Distribution of articles that met search criteria and passed filters (*n* = 53) by publication year

The results of the literature search and selection process are shown in Fig. 1. In brief, our PubMed search filtered for language and species resulted in 88 articles, with a publication range between 2017 and 2025 (Fig. 2) [4–64].

The articles identified from our search are summarized in Table S1 [see Additional file 1]. We applied four further filters: full text availability, whether the study indeed carried out Mendelian randomization, whether the study carried tested vascular cognitive impairment or vascular dementia as the outcome, and whether the sources of the exposure and outcome data were described. Of the 61 identified articles, the full text of three were not accessible via our institution. Another two studies did not carry out Mendelian randomization and two others did not test vascular cognitive impairment or vascular dementia as the outcome in its Mendelian randomization analyses [44, 47, 49, 56]. Finally, for studies that report evidence of a causal link, we removed those that did not explicitly state the source of the exposure and outcome data, which removed an additional study [24]. 53 articles remained after these filters were applied.

The STROBE-MR checklist (https://www.strobe-mr.org; preprinted in 2019 and subsequently published in 2021) was developed to promote transparency and reproducibility of Mendelian randomization studies [65, 66]. We noted that, except for three articles (two published in 2017 [5, 46] and one published in 2020 [6, 49]), the articles that met our filtering criteria were published in 2021 or more recently. Among the articles identified by our systematic review, roughly a third (*n* = 19) included a completed STROBE-MR checklist and/or explicitly stated within the main text that the study adhered to those guidelines.

**Fig. 3:**
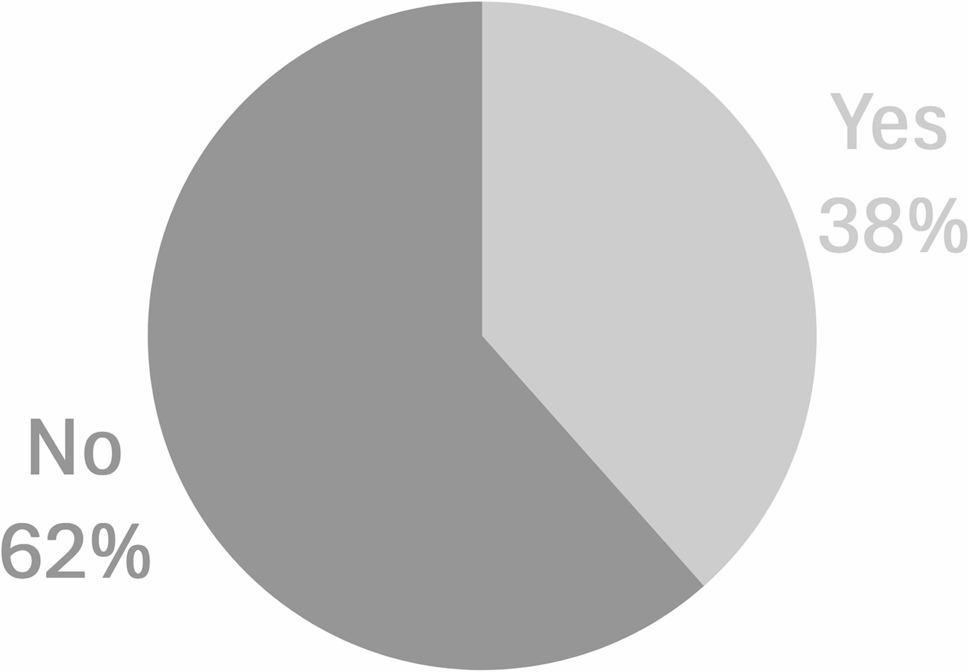
Percentage of articles that reported adherence to the STROBE-MR checklist (“no”, *n* = 16 versus “yes” *n* = 10) among articles that met the search criteria and passed filters that reported a potential causal effect between at least one genetically predicted exposure and vascular dementia (*n* = 26)

Twenty-six of the 53 of the articles (49%) found evidence using two-sample (*n* = 25) or one-sample (*n* = 1) Mendelian randomization for a causal relationship between a genetically predicted exposure and vascular dementia (Table S2) [see Additional file 2]. None of the articles identified in our review assessed vascular cognitive impairment. Only 10 of the 26 articles (38%) suggesting a causal link included a completed STROBE-MR checklist and/or explicitly stated within the main text that the study adhered to those guidelines (Fig. 3). Caution is warranted when interpreting the results.

We summarized the genetically predicted exposures with or without evidence of a causal link with vascular dementia based on the publications assessed in our systematic review in Table S3 [see Additional file 3]. Among the studies that investigated similar exposures, some consistently yielded positive causal results between certain types of exposure and vascular dementia. For instance, a positive link between genetically predicted markers of inflammation and vascular dementia was reported in three distinct studies, which tested as a genetically predicted exposure, C-reactive protein levels, total white blood-cell count, and plasma cortisol levels, respectively (OR [95% CI] = 2.99 [1.53–5.84]; *p* < 0.01 for C-reactive protein levels adjusted for BMI; 1.198 [1.050–1.367]; *p* = 0.007 for total white blood-cell count, and 2.02 [1.00-4.05]; *p* = 0.049 for plasma cortisol levels) [31, 40, 48]. Genetically predicted high neutrophil count, which can be observed in bacterial and viral infections, was not, however, causal for vascular dementia based on a Mendelian randomization study by Luo et al. [41]. Atherosclerotic vascular disease, which was assessed as stroke in one Mendelian randomization and atrial fibrillation (a risk factor for stroke) in another study from our review, was also reported as having a causal effect (increased risk) on vascular dementia (OR [95% CI] = 2.056 [1.049–1.530]; *p* = 0.013 and 1.35 [1.08–1.695]; *p* = 0.01, for stroke and atrial fibrillation, respectively) [12, 33]. Additionally, two Mendelian randomization studies found that genetically predicted psychiatric disorders, specifically, major depressive disorder (MDD) tested as an exposure in one study and delirium tested as an exposure in the other publication, increased risk of vascular dementia (OR [95% CI] = 2.131 [1.249–3.639]; *p* = 0.006 and 1.246 [1.075–1.444]; *p* = 0.003, respectively) [7, 22]. Similarly, a Mendelian randomization study published after our PubMed search had been conducted, reported a causal effect between myalgia (a common comorbidity of MDD) and vascular dementia [67]. Finally, a positive link between type 2 diabetes (OR [95% CI] = 1.11 [1.07–1.15]; *p* = 3.63 × 10^− 9^ and 1.11 [1.04–1.19]; *p* = 2.20 × 10^− 3^ in non-hispanic White and non-hispanic Black datasets, respectively) [36] and diabetic retinopathy (OR [95% CI] = 1.68 [1.01–2.82]; *p* = 0.046) [10] was identified with vascular dementia based on our review of Mendelian randomization studies, whereas work by Benn et al. [6] demonstrated that high plasma glucose increased the risks of unspecified dementia but neither Alzheimer’s disease nor vascular dementia.

In contrast, other publications investigated within our review that assessed related exposures, consistently did not find evidence of causal effects between the exposure of interest and vascular dementia. For example, regarding kidney health, chronic kidney disease was analyzed as an exposure in one Mendelian randomization study included in our review [30], and there was no evidence of a causal link with vascular dementia. In work by Olsson et al. that tested vitamin D (for which its metabolism is highly dependent on kidney function) as an exposure on vascular dementia risk in community-dwelling men in Sweden aged 69 to 74 years similarly did not find evidence of a causal effect [46]. Furthermore, certain neurological comorbidities such as dyslexia [62] and migraines [13, 60] did not show evidence of a causal effect on vascular dementia based on the studies assessed in our review. There was also no significant relationship found between auto-immune disorders such as psoriasis vulgaris [59], lupus [29], and inflammatory bowel disease [37] and vascular dementia. Given the finding in other Mendelian randomization of evidence of a causal effect between genetically predicted levels of inflammation elevated levels of inflammatory markers, such as C-reactive protein, an important marker of inflammation often seen in auto-immune disorders, and vascular dementia [48], further research is warranted into investigating potential causal effects between immune-related markers or states and vascular dementia.

Finally, other Mendelian randomization studies assessing similar exposures reported differing effects depending on the study, resulting in ambiguous conclusions on the exposure-outcome relationship. Cholesterol levels, for instance, was analyzed as an exposure on vascular dementia in multiple studies in our review. One publication found that elevated low density lipoprotein (LDL)-cholesterol and total cholesterol levels increased risk of vascular dementia (OR [95% CI] = 1.94 [1.48–2.55]; *p* = 1.61 × 10^− 6^ and 1.77 [1.32–2.38]; *p* = 1.39 × 10^− 4^, respectively) [26], while another showed that low LDL-cholesterol levels, driven by *PCSK9* and *HMGCR* variants (genes implicated in LDL-cholesterol metabolism), had no causal effect on vascular dementia [5]. This finding is contradicted by a study carried out by Zhang et al. that reported a protective effect of decreased LDL-cholesterol levels mediated by *HMGCR* on vascular dementia albeit with a very wide confidence interval (OR [95% CI] = 18.38 [2.09-161.47]; *p* = 0.009) [58]. Similarly, He et al. [21] tested genetically predicted metabolic syndrome (defined as at least three of the following conditions: abdominal obesity, high blood pressure, high blood sugar, high serum triglycerides, and low serum high-density lipoprotein cholesterol) as the exposure and did not find a causal effect with vascular dementia.

The relationships between vision-related traits or atopic dermatitis on vascular dementia were also inconclusive among the assessed studies. One study found that cataracts was positively linked with vascular dementia (OR [95% CI] = 1.92 [1.26–2.92]; p-value not specified) [17], whereas another study assessing unspecified vision impairment as the exposure did not find a causal effect [28]. Furthermore, two of the publications from our review tested genetically predicted atopic dermatitis as an exposure, and one reported a protective effect of atopic dermatitis on vascular dementia (OR [95%CI] = 0.89 [0.81–0.99]; *p* = 0.031) [53], while the one did not find evidence of a causal relationship [20].

Other studies in our review assessed various types of gut microbiota and metabolites on vascular dementia risk using Mendelian randomization. Some types of microbiota or metabolites showed evidence that increased levels had a causal effect of (either increasing risk or decreasing risk) on vascular dementia, while others had no effect [25, 26, 32].

Finally, four studies investigated in our review performed bidirectional Mendelian randomization, testing vascular dementia as either the outcome or as the exposure, and found evidence for a causal relationship between vascular dementia as the exposure and at least one tested outcome (Table S4) [see Additional file 4]. In summary, genetically predicted vascular dementia was reported as having a potential causal relationship with migraine [13], increased or decreased levels of certain immune-cell phenotypes [11], increased levels of seven circulating cytokines [55], and decreased levels of high-density lipoprotein cholesterol [58]. These results were derived using genome-wide association study summary statistics of European genetic ancestry through inverse variance weighted two-sample Mendelian randomization.

## Discussion

Despite calls from the Mendelian randomization community for robust studies [65, 66, 68, 69], we found that only roughly a third of studies analyzed within the context of our systematic review stated that their work adhered to the STROBE-MR checklist. Following the STROBE-MR guidelines does not necessarily guarantee a robust Mendelian randomization study, but it is a minimal step to promote reproducible and transparent Mendelian randomization efforts.

None of the studies identified in our review assessed vascular cognitive impairment; all investigated causal effects of genetically predicted exposures on vascular dementia. Of the exposures tested in the 53 articles looked into for this review Table S3 [see Additional file 3], known risk factors for vascular dementia, depression [22], stroke [12], diabetes [36], atrial fibrillation [33], all demonstrated a significant causal effect with vascular dementia, where increased levels of the genetically predicted exposure increases vascular dementia risk. These results demonstrate the ability of Mendelian randomization to identify causal relationships.

To achieve sufficient statistical power, given the prevalence of vascular dementia in the general population, large sample sizes are required in genetic association analyses (which are subsequently used for two-sample Mendelian randomization). All but one of the publications assessed in this review that presented evidence of a potential causal relationship between an exposure and vascular dementia utilized two-sample (summary-level) Mendelian randomization based on genetic variant-trait association data for vascular dementia derived from individuals of European genetic ancestry. Two-sample Mendelian randomization can be a useful strategy to increase power when individual data are not available or if a single cohort does not have large sample sizes for the both the exposure and outcome of interest. All of the 25 articles presenting evidence of a potential causal effect through two-sample Mendelian randomization specified one of two sources for the vascular dementia outcome: the Finland-based FinnGen Biobank [70] (*n* = 21) or the United Kingdom-based UK Biobank [71] (*n* = 4). Both cohorts are large population-based biobanks of around half a million individuals each (or aiming for that sample size in the case of FinnGen), with both genetic data and a wealth of phenotypic data, including International Classification of Diseases (ICD-9 and ICD-10) codes from health registries, through which vascular dementia cases can be identified. FinnGen has the greater number of vascular dementia cases, ~ 1000–2000 depending on the version, whereas UK Biobank has only ~ 400 vascular dementia cases.

The one-sample Mendelian randomization study was carried out in the Million Veterans Program cohort and tested for causal effects between type 2 diabetes in three groups: non-Hispanic white, non-Hispanic black and Hispanic, finding evidence of a causal link with vascular dementia in the first two groups with similar effects [36]. Investigations in individuals of other genetic ancestries is warranted to assess the transposability of results.

Our findings have implications for future research. First, we join voices with the community for carrying out robust Mendelian randomization studies supported by multiple lines of evidence and adhering to reporting guidelines (e.g. STROBE-MR). Studies for which we identified that demonstrated contradictory results could be due to numerous reasons, including invalidity of certain Mendelian randomization assumptions particularly if STROBE-MR guidelines were not adhered to, non-generalizability of results from one study population to another, or differential or non-existent modes of accounting for multiple testing in studies that tested multiple exposure-outcome pairs.

Second, we advocate for the need to carry out genetic epidemiological approaches in individuals of non-European genetic ancestry, as data becomes available. For instance, almost half of the large All of Us Research Program cohort are from historically underrepresented minorities. Through the availability of genetic data and a wealth of phenotypic data, this cohort, for example, assuming robust analytical approaches and careful phenotype definitions, offers a potential resource for one-sample or two-sample Mendelian randomization analyses to replicate findings and to begin to assess transposability of results in individuals of non-European genetic ancestry [72]. We highlight the call for replication in cohorts of African, Asian, Latin American identifiers.

Third, we suggest further investigation of the risk factors (i.e. exposures) identified as having potential causal effects for vascular dementia, which could open new avenues for prevention or treatment.

To conclude, in our systematic review, we summarized evidence from Mendelian randomization studies of genetically predicted exposures on vascular dementia risk. Some exposures presented evidence of a causal effect in at least one study in our review, including known risk factors. Potential causal risk factors were reported for a variety of exposures, such as higher C-reactive protein levels [48], higher plasma cortisol levels [31], decreased iron load in the brain [52] and liver fibrosis and cirrhosis [34]. However, independent evidence from other study designs (meaning employing a triangulation approach by gathering evidence from longitudinal cohorts, randomized trials and other designs) in addition to testing for effects in other populations are needed for eventual actionable implementations of the exposures of interest on vascular dementia.

## Supplementary Information

Additional file 1: Table S1. Overview of published articles from the literature review.

Additional file 2: Table S2. Summary of articles presenting evidence of a causal relationship between an exposure and vascular cognitive impairment (VCI) or vascular dementia (VD) using Mendelian randomization (MR).

Additional file 3: Table S3. Overview of genetically predicted exposures with or without evidence of influencing vascular dementia (VD) based on the systematic review.

Additional file 4: Table S4. Summary of articles presenting evidence of a causal relationship between vascular cognitive impairment (VCI) or vascular dementia (VD) and an exposure using Mendelian randomization (MR).

Additional file 5: PRISMA checklist.

## Data Availability

Not applicable.
